# Lithium Prescribing during Pregnancy: A UK Primary Care Database Study

**DOI:** 10.1371/journal.pone.0121024

**Published:** 2015-03-20

**Authors:** Rachel L. McCrea, Irwin Nazareth, Stephen J. W. Evans, David P. J. Osborn, Vanessa Pinfold, Phil J. Cowen, Irene Petersen

**Affiliations:** 1 University College London, Department of Primary Care and Population Health, London, United Kingdom; 2 London School of Hygiene and Tropical Medicine, London, United Kingdom; 3 University College London, Division of Psychiatry, London, United Kingdom; 4 The McPin Foundation, London, United Kingdom; 5 University of Oxford, University Department of Psychiatry, Oxford, United Kingdom; University of Rennes-1, FRANCE

## Abstract

**Background:**

Women taking lithium must decide whether to continue the medication if they conceive or plan to conceive. Little is known about the extent of prescribing of lithium during pregnancy.

**Aims:**

To determine: 1) the prevalence of lithium prescribing during pregnancy and 2) to assess whether pregnancy is associated with discontinuation of lithium.

**Method:**

First, we identified women receiving any lithium prescriptions before and during pregnancy using The Health Improvement Network (THIN) primary care database. Subsequently, we used a Kaplan-Meier plot to compare time to last prescription in women prescribed lithium continuously three months before pregnancy and a comparison group of non-pregnant women. Finally, we described the characteristics of the women prescribed lithium in pregnancy.

**Results:**

Very few women were prescribed lithium during pregnancy; out of 458,761 pregnancies, we identified 47 (0.01%) in which lithium was prescribed after the 6th week of pregnancy (when the pregnancy was likely to be known). In our study of discontinuation, we found pregnant women were more likely to stop lithium than those who were not pregnant. Of the 52 women who were being continuously prescribed lithium three months before pregnancy, only 17 (33%) continued receiving prescriptions beyond the 6th week of pregnancy. However, most of these 17 women continued treatment throughout pregnancy.

**Conclusions:**

Pregnancy was strongly associated with discontinuation of lithium. Further evidence on the risks of lithium is needed so that women can weight these against the risk of a deterioration in maternal mental health.

## Introduction

Lithium is an effective drug for the treatment of bipolar disorder and treatment resistant depression. Women who are taking lithium face a difficult decision about whether or not to stop their medication if they conceive or plan to conceive. Lithium has been linked in the past to an increased risk of congenital cardiac abnormalities, and in particular Ebstein’s anomaly.[1,2] More recent studies have failed to find statistically significant evidence of an association,[3] but the small numbers of women taking lithium in all of these studies mean that considerable uncertainty remains. This potential risk of harm to the unborn baby must be balanced against the increased risk of relapse if the mother stops her medication during pregnancy,[4] which may cause great distress with the potential for adverse outcomes in both mother and baby.

The National Institute for Health and Care Excellence (NICE) 2014 guideline on antenatal and postnatal mental health advises that lithium should not be offered to women who are planning pregnancy or are pregnant unless antipsychotic medication has not been effective.[5] If a woman becomes pregnant while taking lithium, the guideline recommends considering gradually stopping lithium over 4 weeks if the woman is well. For women who are not well or are at high risk of relapse, recommended options include switching gradually to an antipsychotic, stopping lithium and restarting in the second trimester or continuing lithium if an antipsychotic is unlikely to be effective. However, there is little information available about what proportion of women who are taking lithium before pregnancy actually stop their medication when they become pregnant. The aims of this study were to investigate the prevalence of lithium prescribing during pregnancy and to explore discontinuation of lithium during pregnancy. We also examined how many women restarted lithium during and after pregnancy as well as the prescribing of alternative medications during this period.

## Methods

### Data

The data for this study come from The Health Improvement Network (THIN) (http://csdmruk.cegedim.com/), a UK primary care database containing the electronic health records of approximately 12 million patients who have received care from a general practitioner (GP, or family doctor). THIN is broadly generalisable to the UK population in terms of demographics and prevalence of major diseases.[6] The database contains records of all prescriptions issued by a GP—most prescribing in the UK is performed by GPs, even when the decision to initiate a particular drug is made by a hospital specialist.

For this study, we identified women who were pregnant between 1^st^ January 1995 and 31^st^ December 2012. We calculated the start dates and durations of pregnancies using the last menstrual period date, antenatal and postnatal care records, delivery information and records of gestational age at birth; where there was no information on the duration of pregnancy, this was assumed to be 280 days. The pregnancy start date was defined as the first day of the woman’s last menstrual period. Women were required to be permanently registered with the same GP practice throughout the pregnancy and for 6 months beforehand. We excluded pregnancies with records indicating miscarriages and terminations since there was limited information with which to estimate the pregnancy start date for these women. The resulting cohort comprised 458,761 pregnancies.

First, we identified women receiving any lithium prescription in the 6 months before pregnancy, during pregnancy (at any time) and during pregnancy (after the first 6 weeks).

Women were included in the lithium discontinuation study if they were being prescribed lithium continuously 91 days before the start of pregnancy; this was defined as receiving at least one lithium prescription during the 183–92 days before the start of pregnancy, followed by at least one further prescription during the 91–1 days before the start of pregnancy (and no more than 91 days after the preceding prescription). We included prescriptions for lithium carbonate and lithium citrate (chapter 4.2.3 of the British National Formulary). We considered a prescription to be the last in a period of prescribing if there were no subsequent prescriptions issued in the following 91 days. The majority of women received prescriptions that lasted for one or two months. To allow room for women to return to receive a repeat prescription earlier or later we set the cut-off at 91 days.

One woman had two eligible pregnancies and we randomly selected one of these for inclusion.

We also selected a comparison group of twice as many non-pregnant women prescribed lithium. To do this, we identified ‘non-pregnant’ periods between 1^st^ January 1995 and 31^st^ December 2012 for all women aged 14–50 included in THIN: these were at least 1 year after the end of any previous pregnancy and a minimum of 2 years before any subsequent pregnancy. We randomly selected an index date within these non-pregnant periods to simulate the pregnancy start date. We then identified women who were being prescribed lithium 91 days before the index date, and randomly sampled 2 non-pregnant women for each pregnant woman selected earlier, stratified within 5 year age bands.

### Analysis

First, we described the prevalence of lithium prescribing before and during pregnancy, stratified by 3 year calendar periods.

For our study on discontinuation, we then used the cohort of women who were continuously prescribed lithium before pregnancy and analysed time to last prescription of lithium using Kaplan-Meier survival curves: follow-up started at 91 days before the start of pregnancy, and was censored either at 220 days after the start of pregnancy (two months before a full-term delivery) or at the actual date of delivery for premature births before this date. We described the characteristics of the women who continued treatment after 6 weeks and those who stopped, but did not attempt further statistical analyses because of the small numbers prescribed lithium before pregnancy. We compared the two groups on age, prior continuous time on lithium, parity and other medication prescribed in the 183–92 days before pregnancy—where parity was not recorded in the mother’s medical record, we estimated parity using the number of previous deliveries and older children in the household. Finally, we identified women who restarted lithium prescriptions during and after pregnancy, as well as those prescribed alternative medications. The data were analysed in Stata version 12.1 for PC.

### Ethics statement

Ethical approval for the use of anonymised patient data from THIN for scientific research was provided by the National Health Service South-East Multicentre Research Ethics Committee (MREC) in 2002. This study was approved by the Scientific Review Committee of CSD Medical Research in September 2013.

## Results

### Prevalence of lithium prescribing in pregnancy

From the cohort of 458,761 pregnancies, we identified only 67 pregnancies (0.015%) in which the woman received one or more prescriptions of lithium at any time during pregnancy. However, this included only 47 (0.010%) pregnancies in which the woman received further prescriptions after six weeks, at which point the woman was likely to be aware of the pregnancy. The prevalence of lithium prescribing before and during pregnancy remained very low between 1995 and 2012 (Table 1).

**Table 1:** Prevalence of lithium prescribing to pregnant women over time, before and during pregnancy.

Period	Prescribed in 6m before pregnancy		Prescribed during pregnancy (any time)		Prescribed during pregnancy (after first 6 weeks)		Total pregnancies
	n	%	n	%	n	%	n
1995–1997	8	0.019	8	0.019	8	0.019	42,239
1998–2000	17	0.026	10	0.015	8	0.012	65,955
2001–2003	17	0.022	12	0.016	7	0.009	75,806
2004–2006	18	0.021	13	0.015	6	0.007	87,262
2007–2009	17	0.018	10	0.011	7	0.007	94,617
2010–2012	17	0.018	14	0.015	11	0.012	92,882
**All years**	**94**	**0.020**	**67**	**0.015**	**47**	**0.010**	**458,761**

### Discontinuation of lithium before and during pregnancy

**Fig 1:**
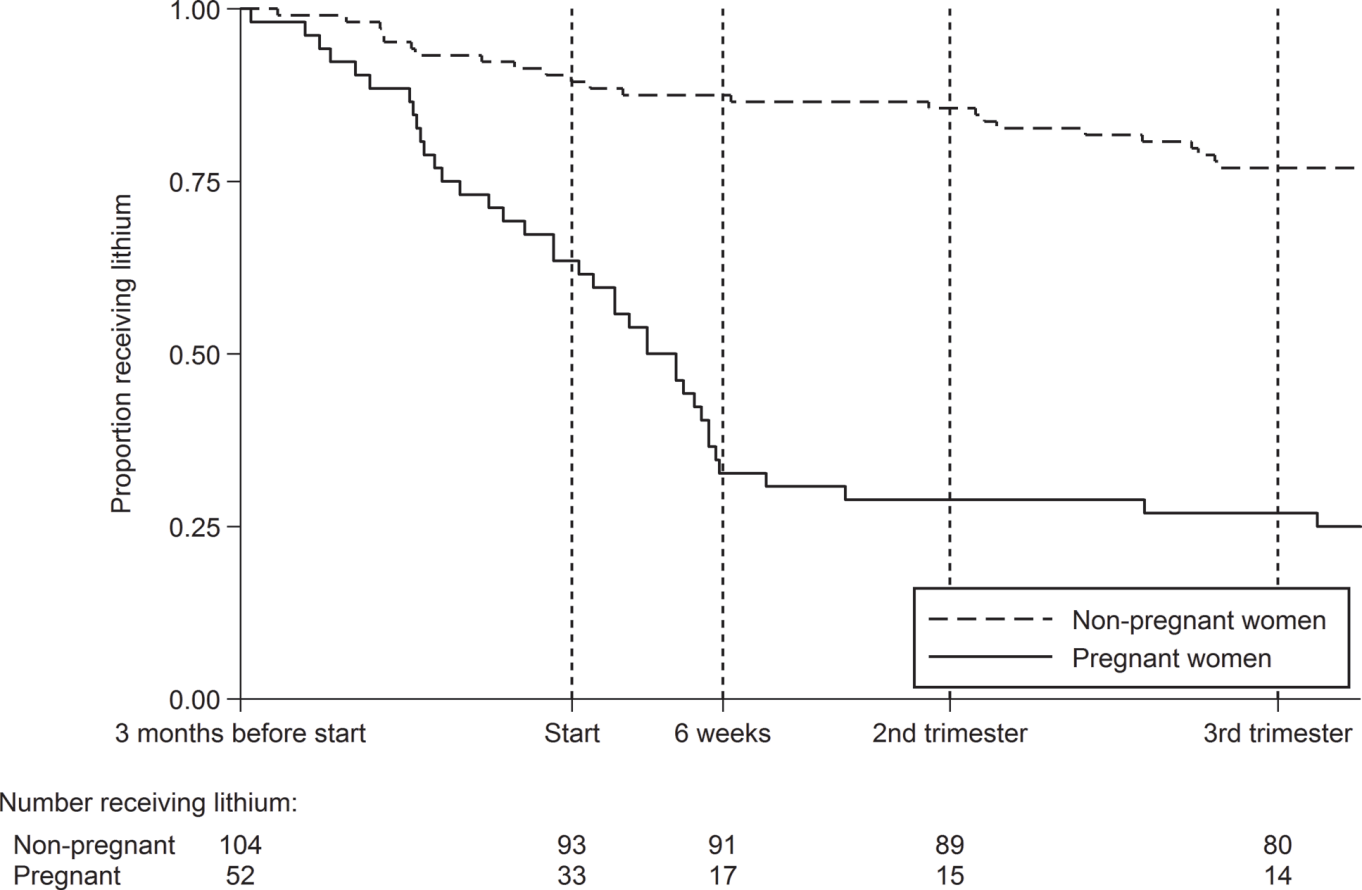
Discontinuation of lithium in pregnant and non-pregnant women.

In our discontinuation cohort, we identified 52 women who were being prescribed lithium continuously 91 days before the start of their pregnancy. Of these women, many discontinued treatment either before or in the first few weeks of pregnancy (Fig. 1). Only 33% (17 women) continued to receive prescriptions after the 6^th^ week of pregnancy (by which time they were likely to be aware that they were pregnant). However, 15 out of these 17 women continued to receive prescriptions throughout pregnancy. Table 2 compares some of the characteristics of those who continued to receive prescriptions beyond 6 weeks with those who stopped before this date. A greater proportion of those who continued had been prescribed an antidepressant (47%) or antipsychotic (53%) in addition to lithium during the 183–92 days before pregnancy (compared to 34% prescribed antidepressants or antipsychotics in those who stopped). In addition, a greater proportion of those who continued lithium were having their first child (59% versus 40%) or had been receiving continuous lithium prescriptions for less than 6 months (47% versus 31%). However, the small numbers of women involved mean that the confidence intervals for these percentages are wide and generally overlap (see Table 2).

**Table 2:** Comparing the characteristics of those who continue to receive lithium prescriptions beyond 6 weeks and those who stop.

	Stopped before 6 weeks (n = 35)			Continued beyond 6 weeks (n = 17)		
	n	%	(95% CI)	n	%	(95% CI)
**Age band**						
<25	5	14.3	(5.9, 30.8)	0	0.0	-
25–29	8	22.9	(11.5, 40.2)	4	23.5	(8.6, 50.1)
30–34	10	28.6	(15.7, 46.2)	7	41.2	(20.2, 66.0)
35+	12	34.3	(20.2, 51.9)	6	35.3	(16.0, 60.9)
**Prior continuous time on lithium**						
<6 months	11	31.4	(17.9, 49.0)	8	47.1	(24.5, 70.8)
6–12 months	6	17.1	(7.7, 34.0)	2	11.8	(2.7, 38.8)
>12 months	18	51.4	(34.7, 67.8)	7	41.2	(20.2, 66.0)
**Estimated parity**						
0	14	40.0	(24.8, 57.4)	10	58.8	(34.0, 79.8)
1	13	37.1	(22.5, 54.6)	4	23.5	(8.6, 50.1)
2	6	17.1	(7.7, 34.0)	3	17.6	(5.4, 44.4)
3 or more	2	5.7	(1.4, 21.1)	0	0.0	-
**Also taking an antidepressant**	12	34.3	(20.2, 51.9)	8	47.1	(24.5, 70.8)
**Also taking an antipsychotic**	12	34.3	(20.2, 51.9)	9	52.9	(29.2, 75.5)
**Also taking an anticonvulsant**	6	17.1	(7.7, 34.0)	3	17.6	(5.4, 44.4)

Notes: Prior continuous time on lithium was measured up to the 91^st^ day before the start of pregnancy. Antidepressants, antipsychotics and anticonvulsants were recorded in the 183–92 days before the start of pregnancy. Where parity was not recorded in the mother’s medical records, we also looked at numbers of previous deliveries and older children in the household.

Of the 39 pregnant women who stopped lithium prescriptions in Fig. 1, only 7 received prescriptions for an antipsychotic or anticonvulsant mood stabiliser in the three months after discontinuing lithium. Four of the 39 women restarted lithium prescriptions during pregnancy. Many more restarted after delivery; six months after delivery, 57% of the original cohort were receiving prescriptions for lithium (29/51, with 1 woman lost to follow-up after delivery), and the proportion receiving prescriptions remained stable beyond this point.

### Comparison with discontinuation of lithium in women who are not pregnant

Women may discontinue lithium for many reasons even when they are not pregnant. To illustrate this, Fig. 1 also shows the time to last prescription in a group of women who were not pregnant: 88% (91 out of 104) were still receiving continuous prescriptions 6 weeks after the randomly selected index date, while 77% (80 out of 104) were still receiving prescriptions at the end of the follow-up period illustrated.

## Discussion

We found that pregnancy is a strong predictor of lithium discontinuation in women who were being prescribed the drug before they became pregnant. Only 33% of women who were being prescribed lithium 3 months before the start of pregnancy received further prescriptions after the 6^th^ week of pregnancy. Most of those who discontinued were not prescribed an alternative mood stabiliser or antipsychotic and few restarted lithium during pregnancy. However, 6 months after delivery 57% of the original cohort were being prescribed lithium.

A number of women received their last prescription *before* the start of pregnancy; this suggests that these women stopped taking lithium before they became pregnant. However, we cannot know exactly when women discontinued, particularly since they may have been advised to reduce the dose gradually following their final prescription. We noted that many women had also been receiving antidepressants and antipsychotics alongside lithium prior to pregnancy. It is possible that co-medication is a marker for severity of the underlying illness, although we could not detect a statistically significant difference in co-medication between those who continued lithium and those who stopped.

The pregnant women in our study were much more likely to discontinue lithium than our comparison group. However, women who become pregnant may differ in important ways from those who do not. For example, the severity of their underlying illness may be different. Therefore, we cannot attribute the difference in discontinuation of lithium solely to the pregnancy. However, the fact that many women restarted lithium in the 6 months after delivery suggests that pregnancy did contribute to their stopping.

It seems likely that many of the women who discontinued lithium did so because of concerns around the safety of lithium during pregnancy and breastfeeding. Also, it is plausible that many women on lithium treatment may elect not to become pregnant or terminate the pregnancy for various reasons. This may partly explain why we identified relatively few pregnant women who were prescribed lithium before pregnancy. It is also possible that for various reasons lithium is now being used less in the management of bipolar disorders and other mood stabilisers and antipsychotic drugs are being preferred. However, the teratogenic risks of lithium may have been overstated.[3] Furthermore, concerns about the potential harm of lithium must be weighed against the risk of deterioration in maternal mental health after stopping lithium,[4] and the importance of giving mother and child a stable and secure start to life together.

Other studies on discontinuation of psychotropic medication in pregnancy demonstrate remarkably similar patterns to the ones we found on lithium. Thus, Petersen *et al*. showed that of women prescribed antidepressants prior to pregnancy only 1060/5229 (20%) women continued to receive prescriptions beyond 6 weeks of pregnancy. [7] Man *et al*. found that women with a diagnosis of bipolar disorder or depression prescribed antiepileptic drugs were also highly likely to discontinue prescriptions when they became pregnant and less than 25% continued prescriptions beyond 6 weeks of pregnancy.[8]

Our study highlights a key difficulty in evaluating lithium use and safety during pregnancy: despite using data from a very large primary care database, we still only identified a very small number of women who were prescribed lithium before and during pregnancy. A recent systematic review also identified this as an issue.[3] Another key limitation when using prescribing data is that we do not know whether women actually took the medication that they were prescribed, and for those who discontinued we cannot determine the exact date on which they stopped taking the drug. However, studies that are based on interviews or questionnaires may also face difficulties in achieving exact information on drug usage.

While women who are prescribed lithium in the United Kingdom are most likely to receive this from primary care (since this is where the prescribing budget lies), we cannot rule out the possibility that some women received prescriptions from specialist mental health services during pregnancy (which would not be recorded in electronic primary care records). This may particularly be an issue for women who were admitted to hospital following a relapse. Thus, we may have slightly over-estimated the level of discontinuation.

We found the prevalence of lithium prescribing during pregnancy was very low. The majority of women who were prescribed lithium 3 months before becoming pregnant stopped receiving prescriptions by the 6^th^ week of pregnancy. Decisions about medication during pregnancy will always require weighing up the benefits and risks, but such judgements are made much harder when the risks are so uncertain. This highlights the urgent need for further research into the safety of lithium in pregnancy and the need for unbiased reporting of both positive and negative results, even if each small study can only contribute a small piece to the overall puzzle.
